# 1434. Treatment Patterns, Healthcare Resource Use, and Associated Costs in Females with Uncomplicated Urinary Tract Infection in the United States

**DOI:** 10.1093/ofid/ofab466.1626

**Published:** 2021-12-04

**Authors:** Rena Moon, Alen Marijam, Fanny S Mitrani-Gold, Daniel C Gibbons, Alex Kartashov, Ning Rosenthal, Ashish V Joshi

**Affiliations:** 1 Premier Applied Sciences, Premier Inc., Charlotte, NC, USA, Charlotte, North Carolina; 2 GlaxoSmithKline plc., Collegeville, PA, USA, Collegeville, Pennsylvania; 3 GlaxoSmithKline plc, Collegeville, PA, USA, Chicago, Illinois; 4 GlaxoSmithKline plc., Brentford, Middlesex, UK, Brentford, England, United Kingdom

## Abstract

**Background:**

Urinary tract infections (UTIs) disproportionately affect women and are a substantial burden on healthcare systems. We assessed the effect of antibiotic (AB) switching on UTI recurrence, healthcare resource use (HRU), and related costs among adolescent and adult females in the US with uncomplicated UTIs (uUTIs).

**Methods:**

This retrospective cohort study used US Optum claims data (United Healthcare, January 1, 2013–December 31, 2018). Eligible patients were females ≥ 12 years of age with an acute uUTI diagnosis at outpatient or emergency department (ED) visit (index date) and an oral AB prescription within ± 5 days of index. Patients with recurrent UTIs (rUTIs), defined as 2 UTI diagnoses (including index) in 6 months or ≥ 3 UTI diagnoses (including index) in 12 months, were included; those with complicated UTI were excluded. Patients were assigned to two groups: AB switch (≥ 2 filled prescriptions of different AB within 28 days post index [uUTI episode]) and no AB switch.

**Results:**

In 5870 eligible patients (mean age 44.5 years; 76.6% White), ciprofloxacin (CIP; 38.6%), nitrofurantoin (NFT; 31.4%), and trimethoprim-sulfamethoxazole (TMP-SMX; 25.6%) were the most commonly prescribed first-line ABs at index, and 567 (9.7%) patients switched AB. CIP was switched to NFT and TMP-SMX in 2.0% and 1.7% of patients, respectively. NFT was switched to CIP and TMP-SMX in 2.6% and 1.5% of patients, respectively. TMP-SMX was switched to CIP and NFT in 3.0% and 2.4% of patients, respectively. During index visit, the AB switch group had higher mean ambulatory care and pharmacy claims (both p < 0.001), and higher total mean HRU costs (&2186.4) per patient compared with the no switch group (&1508.8; p = 0.011). More patients had rUTI in the AB switch group (18.9%) versus the no switch group (14.2%; p < 0.001), and more had ED visits in the AB switch group than the no switch group (p < 0.0001) (Table 1). During follow-up, the AB switch group had a higher mean number of uUTI episodes per patient (p < 0.001; Table 1), and more patients had UTI-related ED visits (10.8%) compared with the no switch group (7.7%; p = 0.010; Table 2).

Table 1. Primary outcomes of uncomplicated UTI outpatients during January 1, 2013–December 31, 2018, stratified by any switch in AB use during index episode

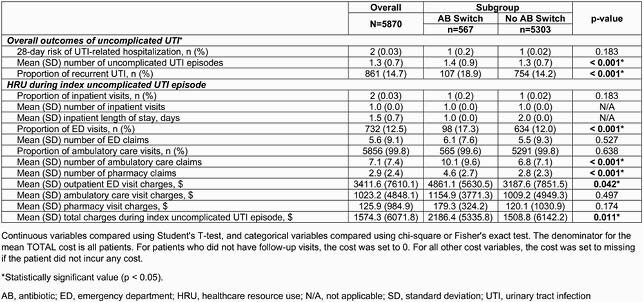

Table 2. Primary outcomes of uncomplicated UTI outpatients during January 1, 2013–December 31, 2018, stratified by any switch in AB use during 12-month follow-up

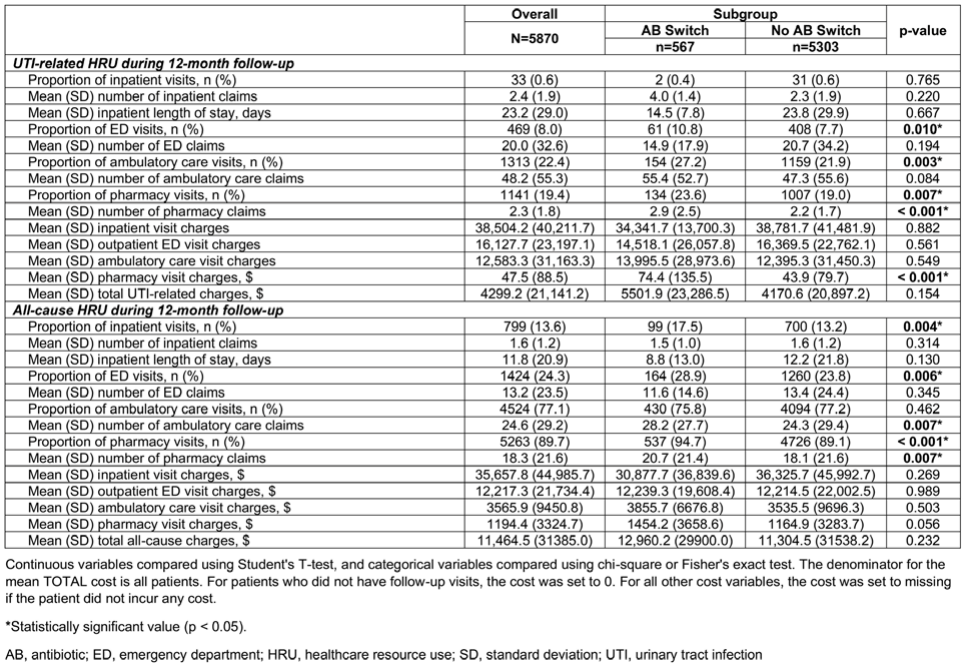

**Conclusion:**

US females with uUTI who switched AB had more rUTI cases and increased overall costs and HRU compared with those who did not switch AB, suggesting an unmet need for improved prescribing practices.

**Disclosures:**

**Rena Moon, MD**, **Premier Applied Sciences, Premier Inc.** (Employee) **Alen Marijam, MSc**, **GlaxoSmithKline plc.** (Employee, Shareholder) **Fanny S. Mitrani-Gold, MPH**, **GlaxoSmithKline plc.** (Employee, Shareholder) **Daniel C. Gibbons, PhD**, **GlaxoSmithKline plc.** (Employee, Shareholder) **Alex Kartashov, PhD**, **Premier Applied Sciences, Premier Inc.** (Employee) **Ning Rosenthal, MD**, **Premier Applied Sciences, Premier Inc.** (Employee, Shareholder) **Ashish V. Joshi, PhD**, **GlaxoSmithKline plc.** (Employee, Shareholder)

